# Supplementary material for: The Impact of COVID-19 on Nursing Homes: Study Design and Population Description
Source: Int J Environ Res Public Health. 2022 Dec 10;19(24):16629. doi: 10.3390/ijerph192416629 (PMC9779101; doi:10.3390/ijerph192416629)
Supplement: Supplementary file 1 [file ijerph-19-16629-s001.zip › Supplemenraet Table S1.pdf]

Table S1. Measures included in the COVID-19 Residential Homes Survey (Madrid Region, Spain).

Personal circumstances regarding the pandemic			
**Areas studied and measures used**	**Items**	**Response categories**	**Scale variables and minimum and maximum values Cronbach Alpha**
Coronavirus status		1 = I have not had the disease<br>2 = I have had the disease, but I am still suffering after-effects<br>3 = I have had the disease, but I do not have any after-effects	
If they have had coronavirus and reported after-effects: type and number	Pain; Neurological; Respiratory; Cardiac; Digestive; Tiredness/malaise; Sadness/anxiety	1 = Yes<br>0 = No	Number of effects of having had COVID-19: 1-4
Worried about the COVID-19 pandemic		1 = Not at all<br>2 = Somewhat<br>3 = Quite a lot<br>4 = A lot<br>999 = Do not know/No answer (DK/NA)	
Satisfaction with life in compared perspective (before and during the pandemic)		1 = Less<br>2 = The same<br>3 = More<br>999 = DK/NA	
Fear of developing COVID-19, according to the FCV-19S scale	1. I am very afraid of COVID-19<br>2. Thinking about COVID-19 makes me uncomfortable<br>3. My palms sweat when I think about COVID-19<br>4. I feel afraid of losing my life because of COVID-19<br>5. I get nervous or anxious when I see news and stories about COVID-19<br>6.I can't sleep from worrying about getting COVID-19<br>7. My heart starts beating fast or I feel palpitations when I think about getting COVID-19	1 = Strongly disagree<br>2 = Disagree<br>3 = Neither agree nor disagree<br>4 = Agree<br>5 = Strongly agree<br>999 = DK/NA	Fear scale: 7-35<br>Interpretation: higher values=more fear<br>Cronbach Alpha: 0.941

Protective measures to keep yourself safe from coronavirus: type and number	1. Use a mask<br>2. Wash my hands<br>3. Keep a physical distance<br>4. Stay in my room as much as possible<br>5. Stay away from organized activities in the residential home<br>6. Avoid physical contact with other residents<br>7. Ventilate my room, stay in ventilated places<br>8. Others<br>9. None of these measures	1 = Never<br>2 = Sometimes<br>3 = Often<br>4 = Always<br>999 = DK/NA	Number of measures followed: 1-4

**Health**			
**Areas studied and measures used**	**Items**	**Response categories**	**Scale variables and minimum and maximum values**<br>**Cronbach Alpha**
Resident's previous pathologies from medical reports	Presence of 21 health conditions and an open option of "other"	1 = Yes<br>0 = No	Number of pathologies diagnosed: 1-12
Intake of medications from medical reports	8 groups of medications	1 = Yes<br>0 = No	Number of medications taken: 1-8
Self-assessment of health status		1 = Very poor<br>2 = Poor<br>3 =Fair<br>4 = Good<br>5 = Very good	
Diagnosis of COVID-19 by a health professional		1 = Yes<br>0 = No<br>999 = DK/NA	
If diagnosed by COVID-19: if hospitalized and number of days in hospital		1 = Yes<br>0 = No	If hospitalized: No of days: 3-45
Abbreviated Geriatric Depression Scale	Positive item<br>1. Are you basically satisfied with your life?<br>Negative items<br>4. Are you often bored?<br>9. Do you prefer to stay in residence or in your room instead of going out and doing new things?	Yes<br>No	Depression scale: 0-5<br>Interpretation: Higher values = more depression<br>0-1: no depression;<br>≥ 2: suspicion of depression (mild, moderate, severe)<br>Cronbach Alpha: 0.207

	12. Do you feel useless the way you are now?<br>14. Do you think your situation is hopeless?		
How often he/she feels lonely		1 = Never or hardly ever<br>2 = Seldom<br>3 = Quite often<br>4 = Always or almost always<br>9 = DK/NA	
If feels lonely other than Never or Hardly Ever: Change in perception of loneliness in comparative perspective (before and during the pandemic)		1 = Less lonely<br>2 = The same<br>3 = More lonely<br>999 = DK/NA<br>999 = Does not apply	
Situation regarding use of health care services during pandemic and lockdown	1. Cancellation of a visit to a doctor or other health professional<br>2. Face-to-face visit to a physician or other health care professional<br>3. Video call consultation with a physician or other health care professional	1 = Yes<br>0 = No	
Satisfaction with the care and health care received in the residential home during the pandemic, or with the resources used	1. Medical staff<br>2. Nursing staff<br>3. Physical therapy/rehabilitation<br>4. Aides/geroculturists/caregivers<br>5. Resources (oxygen tanks, nebulizers, drugs, other resources)	1 = Not at all satisfied<br>2 = Not very satisfied<br>3 = Average<br>4 = Fairly satisfied<br>5 = Very satisfied<br>999 = DK/NA	

**Feelings and coping**			
**Areas studied and measures used**	**Items**	**Response categories**	**Scale variables and minimum and maximum values**<br>**Cronbach Alpha**

Positive and Negative Affect Schedule (PANAS)	5 negative affect items<br>(2- Upset/angry; 3- Afraid; 4- Irritated; 6- Nervous; 9- Uneasy)<br>5 items of positive affect<br>(1- Cheerful; 5- Inspired; 7- Determined; 8- Attentive; 10- Active)	1 = Not at all<br>2 = Somewhat<br>3 = Quite a lot<br>4 = Very	Subscales and Interpretation:<br>Positive feelings: 5-20<br>higher values=higher positive feeling<br>Cronbach Alpha: 0.798<br>Negative feelings: 5-20 higher values=higher negative feeling.<br>Cronbach Alpha: 0.809<br>Balance<br>Difference: -12 to 15<br>positive values=more positive feeling;<br>negative values=more negative feeling.<br>Quotient: 0.33 to 4:<br><1: negative balance;= 1: neutral balance;<br>>1: positive balance
Brief Resilient Coping Scale (BRCS)	1. I look for positive ways to change difficult situations<br>2. Regardless of what happens to me, I believe I can control my reactions<br>3. I believe I can grow positively by coping with difficult situations<br>4. I actively look for ways to overcome setbacks that arise in life	1 = This sentence never happens to me<br>2 = It happens to me sometimes<br>3 = It happens to me frequently<br>4 = It happens to me all the time<br>5 = This statement happens to me very often<br>999 = DK/NA.	Resilience scale: 4-20<br>Interpretation: higher values=better ability to cope with adverse situations.<br>Cronbach Alpha: 0.912

**Assessment of the residential environment and activities performed**			
**Areas studied and measures used**	**Items**	**Response categories**	**Scale variables and minimum and maximum values Cronbach Alpha**
Room availability	1. Single<br>2. Shared with spouse/partner/family member<br>3. Shared with another resident<br>999 = DK/NA		
Where did they spend most of their time during the lockdown	1. Always in my room<br>2. I made limited use of different areas<br>3. Unrestricted use of all spaces in the care home		

If they did not make unlimited use of areas: what activities they did outside their room?	1. Eating<br>2. Going for walks<br>3. Group activities<br>4. I have not left my room<br>997. Other, please specify...		
Valuation of various aspects of life in the residential home	1. Friendships with other residents<br>2. Relationships with nursing home workers<br>3. Relationships with family members<br>4. Relationships with friends<br>5. Getting around easily within the residential home<br>6. The chance to go outside the residential home for different activities<br>7. Their perception of safety in the residential home<br>8. Characteristics and quality of the residential home's amenities; (E.g.: room size and design, natural lighting, noise level, temperature, green areas)<br>9. Having a space for privacy (having a single room or other personal space so as not to be disturbed)<br>10. How well their residential home has managed during the COVID-19 pandemic period	1 = Very badly;<br>2 = Badly;<br>3 =Average;<br>4 = Well;<br>5 = Very well<br>999 = Does not have one /DK/NA	Subscales:<br>Relationships: 4-20<br>Cronbach Alpha: 0.771<br>Residential home aspects: 6-15<br>Cronbach Alpha: 0.722<br>Mobility: 2-10<br>Cronbach Alpha: 0.737<br>Space for privacy: 1-5<br>Interpretation: Higher values=better residential rating
Residential home stay in comparative perspective (before and during the pandemic)		1 = Worse<br>2 = The same<br>3 = Better<br>999 = DK/NA	
Activities performed	1. Reading books, newspapers, magazines<br>2. Doing crossword puzzles, sudoku, playing chess, etc.<br>3. Using devices (computer, tablets, cell phones, etc.), to look for information, chat, etc. on the Internet<br>4. Watching TV, listening to the radio<br>5. Walking or exercising<br>6. Going to the chapel<br>7. Doing crafts (sewing, DIY, knitting, etc.)<br>8. Doing things for others	1 = Never (0 days/year)<br>2 = Yearly (3 days/year)<br>3 = Monthly (12 days/year)<br>4 = Weekly (52 days/year)<br>5 = Daily (365 days/year)	
If you perform the activity: changes in performing	Ditto (above)	1 = I have done it less<br>2 =I have kept it up	

activity in compared perspective between previous situation and pandemic situation		3 =I do it more often<br>999 = DK/NA	
Devices or mobile systems used to maintain relationships with your family or friends	1. Yes, through my own devices<br>2. Yes, through other people's or residential home's devices<br>3. No, I do not have access to those devices<br>998. Don't know, don't know that technology<br>999. Not answered		

**Quality of life**			
**Areas studied and measures used**	**Items**	**Response categories**	**Scale variables and minimum and maximum values<br>Cronbach Alpha**
FUMAT-24 scale adapted for elderly people without severe cognitive impairment in nursing homes	24 items in 8 subscales:<br>Emotional well-being<br>Interpersonal relationships<br>Material well-being<br>Personal development<br>Physical well-being<br>Self-determination<br>Social inclusion<br>Rights	1 = Never or hardly ever<br>2 = Sometimes<br>3 = Often<br>4 = Always or almost always.	(Max-Min.) (Cronbach Alpha)<br>Global FUMAT Scale: (45-95) (0.788)<br>Subscales (3-12): emotional well-being (0.859); interpersonal relationships (0.755); personal development (0.833); physical well-being (0.544); self-determination (0.320); social inclusion (0.408); rights (0.802)<br>Material well-being subscale: 6-12 (0.081)<br><br>Interpretation: higher values=higher level of quality of life.

**Ageism and perception of aging**			
**Areas studied and measures used**	**Items**	**Response categories**	**Scale variables and minimum and maximum values<br>Cronbach Alpha**

Ageism	1. Older people are more responsible and competent than other population groups in following the measures established to cope with the virus<br>2. Older people are a problem, a burden, for health and social services<br>3. Older people make their own decisions freely, as do other population groups<br>4. News about the virus' consequences triggers more emotional impact on older people than on other age groups<br>5. In the event of a shortage of health care resources, the elderly should have the same access as other population groups<br>6. Considering the risk of infection, meeting with older people is more dangerous than with other younger groups	1 = Do not agree at all<br>2 = Agree slightly<br>3 = Agree quite a lot<br>4 = Strongly agree<br>999 = DK/NA	<u>Ageism Scale</u>: 0-24
Self-Perception of Aging Scale	1. As I get older things seem worse to me<br>2. I have as much energy as I did last year<br>3. I feel that as I get older I am less useful<br>4. I'm as happy now as I was when I was young<br>5 Things are getting better as I get older	1 = Yes<br>0 = No	<u>Self-Perception Scale</u>: 0-5<br><u>Interpretation</u>: higher values=better perception<br>Kuder-Richardson coefficient KR20: 0.639

**Socio-demographic features**		
**Areas studied and measures used**	**Items**	**Response categories**
Age in years		61-99
Gender	1. Male<br>2. Female<br>9. Prefer not to say	
Marital status	1. Married, living as a couple<br>2. Separated<br>3. Divorced<br>4. Single<br>5. Widow/er	
Level of education	1. Cannot read or write<br>2. Can read and write, but no primary education<br>3. Primary education<br>4. Secondary education<br>5. Vocational education<br>6. University education<br>7. Other non-regulated studies	

	9. I don't know, or I prefer not to answer	
Number of living children		0-5
Number of living daughters		0-6
Total number of children		0-9

NOTE: For further information about the questionnaire: vicente.rodriguez@cchs.csic.es
