# Supplementary material for: The Impact of COVID-19 on Nursing Homes: Study Design and Population Description
Source: Int J Environ Res Public Health. 2022 Dec 10;19(24):16629. doi: 10.3390/ijerph192416629 (PMC9779101; doi:10.3390/ijerph192416629)
Supplement: Supplementary file 1 [file ijerph-19-16629-s001.zip › Supplemenraet Table S2.pdf]

International Journal of
Environmental Research and Public Health

MDPI

Table S2. Sample descriptive results.

Variables	Frequency and valid cases		Statistic values		
Categories	Num	%	Min	Max	Average
**Socio-demographic features**					
Age (years)	447		61	99	83.8
Sex	447				
Male	165	36.9			
Female	282	63.1			
Marital status	447				
Married, living as a couple	64	14.3			
Separated, Divorced	46	10.3			
Single	110	24.6			
Widow/er	227	50.8			
Leve lof education	447				
Cannot read or write	27	6.0			
Can read and write, but no primary education	152	34.0			
Primary education	108	24.2			
Secondary education, Vocational education	120	26.9			
University education	40	8.9			
Number of living children	447		0	5	0.9
Number of living daughters	447		0	6	0.7
Total number of children	447		0	9	1.6
**Personal circumstances regarding the pandemic**					
Coronavirus status	425				
I have not had the disease	233	54.8			
I have had the disease, but I am still suffering after-effects	44	10.4			
I have had the disease, but I do not have any after-effects	148	34.8			
Type and number if they have had coronavirus and reported after-effects					
Pain	17	38.6			
Neurological	17	38.6			
Respiratory	5	11.4			
Cardiac	4	9.1			
Digestive	5	11.4			
Tiredness/malaise	14	31.8			
Sadness/anxiety	3	6.8			
Others	7	15.9			
Number of after-effects (sequelae) after having been infected by coronavirus	44		1	4	1.7

Diagnosis of COVID-19 by a health professional	438				
Non	273	62.3			
Yes	165	37.7			
If diagnosed by COVID-19, were you hospitalised?	165				
Non	129	78.2			
Years	36	21.8			
If diagnosed by COVID-19 and hospitalized: number of days in hospital	36		3	45	14.9
Worried about the COVID-19 pandemic	443				
Not at al	74	16.7			
Somewhat	102	23.0			
Quite a lot	97	21.9			
A lot	170	38.4			
Fear of developing COVID-19, according to the FCV-19S scale	447		7	35	18.4
Number of protective measures to keep yourself safe from coronavirus	447		0	7	4.0
Satisfaction with life before and during the pandemic (comparative perspective)	443				
Less	191	43.1			
The same	238	53.7			
More	14	3.2			
**Health**					
Resident's previous pathologies (num)	439		1	12	6.1
Intake of medications (num)	439		1	8	3.4
Self-assessment of health status	447				
Very poor/Poor	61	13.7			
Fair	124	27.7			
Good/Very good	262	58.6			
Abbreviated Geriatric Depression Scale (GDS, 5 items)	447		0	5	1.7
Situation regarding use of health care services during pandemic and lockdown					
Cancellation of a visit to a doctor or other health professional	433				
Non	364	84.1			
Yes	69	15.9			
Face-to-face visit to a physician or other health care professional	433				
Non	282	65.1			
Yes	151	34.9			
Video call consultation with a physician or other health care professional	433				

Non	419	96.8			
Yes	14	3.2			
Satisfaction with the general and health care received in the residential home during the pandemic, or with the resources used					
Medical staff	401				
Not satisfied	42	10.5			
Satisfied	359	89.5			
Nursing staff	391				
Not satisfied	36	9.2			
Satisfied	355	90.8			
Physical therapy/rehabilitation	301				
Not satisfied	17	5.6			
Satisfied	284	94.4			
Aides/geroculturists/caregivers	348				
Not satisfied	38	10.9			
Satisfied	310	89.1			
Resources (oxygen tanks, nebulizers, drugs, other resources)	213				
Not satisfied	14	6.6			
Satisfied	199	93.4			
**Sentiments and coping**					
Positive and Negative Affect Schedule (PANAS): Positive affects subscale (PANAS)	447		5	20	11.4
Negative affects subscale (PANAS)	447		5	20	8.7
PANAS Balance (aggregation)	447		-12	15	2.7
PANAS Balance (quotient)	447		0	4	1.5
How often he/she feels lonely	447				
Never or hardly ever	210	47.0			
Seldom	113	25.3			
Quite often	66	14.7			
Always or almost always	58	13.0			
If feels lonely other than Never or hardly ever: Change in perception of loneliness before and during the pandemic (comparative perspective)	237				
Less lonely	1	0.4			
The same	111	46.9			
More lonely	125	52.7			
Brief Resilient Cope Scale (BRCS)	447		4	20	15.9
**Assessment of the residential environment and activities performed**					
Room availability	445				
Single	284	63.8			

Shared with spouse/partner/family member	40	9.0			
Shared with another resident	121	27.2			
Where did they spend most of their time during the lock-down	447				
Always in my room	316	70.7			
I made limited use of different areas	120	26.8			
Unrestricted use of all spaces in the care home	11	2.5			
Valuation of various aspects of life in the residential home (4 subscales)					
Relationships	435		4	20	18.5
Residential traits	437		6	15	14.3
Mobility	423		2	10	8.7
Intimate space	435		1	5	4.7
Stay at nursing home before and during the pandemic (comparative perspective)	429				
Worse	60	14.0			
The same	258	60.1			
Better	111	25.9			
**Activities performed**					
Frequency					
Reading books, newspapers, magazines	447				
Never/Yearly/Monthly	189	42.3			
Weekly/Daily	258	57.7			
Doing crossword puzzles, sudoku, playing chess, etc.	447				
Never/Yearly/Monthly	252	56.4			
Weekly/Daily	195	43.6			
Using devices (computer, tablets, cell phones, etc.), to look for information, chat, etc. on the Internet	447				
Never/Yearly/Monthly	244	54.6			
Weekly/Daily	203	45.4			
Watching TV, listening to the radio	447				
Never/Yearly/Monthly	14	3.1			
Weekly/Daily	433	96.9			
Walking or exercising	447				
Never/Yearly/Monthly	52	11.6			
Weekly/Daily	395	88.4			
Going to the chapel	447				
Never/Yearly/Monthly	258	57.7			
Weekly/Daily	189	42.3			
Doing crafts (sewing, DIY, knitting, etc.)	447				
Never/Yearly/Monthly	292	65.3			
Weekly/Daily	155	34.7			

Doing things for others	447				
Never/Yearly/Monthly	286	64.0			
Weekly/Daily	161	36.0			
Devices or mobile systems used to maintain relationships with your family or friends	439				
Yes, through my own devices	354	80.6			
Yes, through other people's or residential home's devices	36	8.2			
No, I do not have access to those devices	49	11.2			
**Quality of life**					
FUMAT-24 scale adapted for elderly people without severe cognitive impairment in nursing homes. 24 items, 8 subscales					
Emotional well-being	447		3	12	9.57
Interpersonal relationships	447		3	12	11.31
Material well-being	447		6	12	10.68
Personal development	447		3	12	10.42
Physical well-being	447		3	12	9.14
Self-determination	447		3	12	7.51
Social inclusion	447		3	12	9.92
Rights	447		3	12	11.10
FUMAT Global Scores	447		45	95	79.64
**Ageism and Self-Perception of Aging Scale**					
Ageism					
Older people are more responsible and competent than other population groups in following the measures established to cope with the virus	420				
Do not agree at all/Agree slightly	92	21.9			
Agree quite a lot/Strongly agree	328	78.1			
Older people are a problem, a burden, for health and social services	437				
Do not agree at all/Agree slightly	178	40.7			
Agree quite a lot/Strongly agree	259	59.3			
Older people make their own decisions freely, as do other population groups	431				
Do not agree at all/Agree slightly	167	38.7			
Agree quite a lot/Strongly agree	264	61.3			
News about the virus' consequences triggers more emotional impact on older people than on other age groups	423				
Do not agree at all/Agree slightly	353	83.5			
Agree quite a lot/Strongly agree	70	16.5			
In the event of a shortage of health care resources, the elderly should have the same access as other population groups	439				

Do not agree at all/Agree slightly	61	13.9			
Agree quite a lot/Strongly agree	378	86.1			
Considering the risk of infection, meeting with older people is more dangerous than with other younger groups	429				
Do not agree at all/Agree slightly	203	47.3			
Agree quite a lot/Strongly agree	226	52,7			
Self-Perception of Aging Scale	408		0	5	1.91

Shade areas: scales. Num: numbers; Min: minimum; Max: maximun.
